# Supplementary material for: Content-rich biological network constructed by mining PubMed abstracts
Source: BMC Bioinformatics. 2004 Oct 8;5:147. doi: 10.1186/1471-2105-5-147 (PMC528731; doi:10.1186/1471-2105-5-147)
Supplement: Additional File 5 — The original Chilibot query results of the term "long-term potentiation (LTP)" and 22 other terms, limiting the latest references analyzed to the years 1990, 1995, 2000, and 2004. [file 1471-2105-5-147-S5.bz2 › chilibotAdditionalFile5/ltp2000/html/LTP_PKA.html]

 


 **LTP** and **PKA** 
  
Found 56 abstracts in PubMed,  **30 abstracts were retrieved and analyzed**.  


---

 Search Google  |
 PDF files only 
|  EDU domain only 

---

**Interactive relationship** (e.g. stimulation, inhibition, etc)

- **LTP**  is differentially sensitive to CaMKII and  **PKA**  inhibitors depending on the history of the synapse.  Ref: 10879537 Nature, 2000
- Transgenic mice R AB mice that express an inhibitory form of a particular type of regulatory subunit of  **PKA**  type Ialpha showed significantly reduced  **LTP**  in area CA1 of hippocampal slices as compared with slices from wild type mice.  Ref: 11110804 J Neurophysiol, 2000
- ... an inhibitor of  **PKA**  was more effective in blocking four train L  **LTP**  than three train L  **LTP** .  Ref: 10575022 J Neurosci, 1999
- Since Rp cAMPS is a  **PKA**  inhibitor that is not selective for any particular isoform of  **PKA**  and these R AB mice show reduced hippocampal  **PKA**  activity resulting from genetic manipulation of a single isoform of  **PKA**  regulatory subunit, our data support the idea that distinct patterns of synaptic activity can produce different forms of  **LTP**  that significantly engage different isoforms of  **PKA** .  Ref: 11110804 J Neurophysiol, 2000
- Similarly, the  **LTP**  induced by a pairing protocol was decreased by postsynaptic intracellular perfusion of the peptide  **PKA**  inhibitor PKI 6 22 amide.  Ref: 10844013 J Neurosci, 2000
- Since the observed  **LTP**  deficits resembled those previously seen in transgenic mice with reduced hippocampal cAMP dependent protein kinase  **PKA**  activity, we searched for possible strain dependent differences in cAMP dependent synaptic facilitation induced by forskolin an activator of adenylate cyclase and IBMX a phosphodiesterase inhibitor.  Ref: 11067991 J Neurophysiol, 2000
- Maintenance of late phase  **LTP**  is accompanied by  **PKA**  dependent increase in AMPA receptor synthesis.  Ref: 9716131 Nature, 1998
- We suggest that neither PKC nor  **PKA**  is required to induce  **LTP**  at this synapse.  Ref: 11069602 Eur J Neurosci, 2000
- Bath application of the  **PKA**  inhibitor H89 suppressed the early  **LTP**  induced by a single tetanus.  Ref: 10844013 J Neurosci, 2000
- In both pathways the late phase of  **LTP**  is mediated by protein kinase A  **PKA**  and mitogen activated protein kinase MAPK.  Ref: 10964936 J Neurosci, 2000
- **PKA**  activation is also observed in the induction of  **LTP** .  Ref: 10623586 Biochem Biophys Res Commun, 2000
- Prior induction of theta  **LTP**  also occluded the potentiation elicited by chemical activation of  **PKA** .  Ref: 10456066 Learn MemLearn Mem, 2000
- These data demonstrate a role of  **PKA**  in the induction and not the expression of cortical  **LTP**  and suggest that if  **PKA**  is involved in the late phase of  **LTP** , it does not appear to be a persistent activation.  Ref: 9786225 Eur J Neurosci, 1998
- The evidence implicating  **PKA**  has come from bath application of drugs during  **LTP**  induction, an approach that does not distinguish between  **PKA**  activation in the presynaptic or postsynaptic cell.  Ref: 10575019 J Neurosci, 1999
- In contrast, the late phase of theta  **LTP**  required synthesis of proteins and RNA and was blocked by inhibitors of  **PKA** .  Ref: 10456066 Learn MemLearn Mem, 1999
- Our results support the idea of a postsynaptic mechanism maintaining late phase  **LTP** , in which AMPA receptor synthesis is increased as a result of  **PKA**  dependent gene transcription.  Ref: 9716131 Nature, 1998
- The increases in synaptic number and N cadherin levels are dependent on cAMP dependent protein kinase  **PKA**  and protein synthesis, both of which are also required for L  **LTP** .  Ref: 11086998 Neuron, 2000
- We find that synthesis of AMPA receptor subunits is increased three hours after  **LTP**  induction this effect on the synthesis of the AMPA receptor is blocked by inhibitors of  **PKA**  and of transcription.  Ref: 9716131 Nature, 1998
- Long term potentiation  [ **LTP** ]   **LTP**  has several different phases, and there is general agreement that the late phase of  **LTP**  requires the activation of adenylyl cyclase AC and cAMP dependent protein kinase  **PKA** .  Ref: 10844013 J Neurosci, 2000
- The initial or early phase of theta  **LTP**  did not require either protein or RNA synthesis and was independent of cAMP dependent protein kinase  **PKA**  activation.  Ref: 10456066 Learn MemLearn Mem, 2000
- Conversely,  **LTP**  induction in naive synapses and depressed synapses increases phosphorylation of the CaMKII site and the  **PKA**  site, respectively.  Ref: 10879537 Nature, 2000
- These results suggest that NO contributes to L  **LTP**  by stimulating guanylyl cyclase and cGMP dependent protein kinase, which acts in parallel with  **PKA**  to increase phosphorylation of the transcription factor CREB.  Ref: 10575022 J Neurosci, 1999
- Previous work has indicated that cerebellar  **LTP**  induction requires presynaptic Ca influx, stimulation of Ca sensitive adenylyl cyclase, and activation of  **PKA** .  Ref: 10575019 J Neurosci, 1999
- The expression of corticothalamic  **LTP**  is associated with a decrease in paired pulse facilitation PPF and blocked by an inhibitor of the cAMP dependent protein kinase A  **PKA** .  Ref: 10516326 J Neurosci, 1999
- These results show that induction of cerebellar  **LTP**  requires a presynaptic signaling cascade, including Ca influx, stimulation of Ca sensitive adenylyl cyclase, and activation of  **PKA** , and argue against a requirement for postsynaptic Ca signals or their sequelae.  Ref: 10575019 J Neurosci, 1999
- Long term potentiation  [ **LTP** ]   **LTP**  in the hippocampus has an early phase E  **LTP**  that can be induced by one or two train tetanization, lasts approximately 1 hr, and is cAMP dependent protein kinase  **PKA**  and protein synthesis independent and a late phase L  **LTP**  that can be induced by three or four train tetanization, lasts >3 hr, and is reduced by inhibitors of  **PKA**  and of protein or RNA synthesis.  Ref: 10575022 J Neurosci, 1999
- ... tetra burst  **LTP**  requires  **PKA**  isoforms containing the mutant RIalpha subunit.  Ref: 11110804 J Neurophysiol, 2000
- These results indicate that  **PKA**  is important in early  **LTP** , that its locus of action is postsynaptic,.  Ref: 10844013 J Neurosci, 2000
- In particular, theta burst  **LTP**  significantly recruits isoforms of  **PKA**  containing regulatory subunits other than the mutant RIalpha subunit.  Ref: 11110804 J Neurophysiol, 2000
- These results suggest that  **PKA**  and MAPK are critical for the expression of a persistent phase of  **LTP**  in the lateral amygdala.  Ref: 10964936 J Neurosci, 2000
- cAMP dependent protein kinase  **PKA**  is believed to play a critical role in the expression of long lasting forms of hippocampal long term potentiation  [ **LTP** ]   **LTP** .  Ref: 11110804 J Neurophysiol, 2000

**Parallel relationship** (e.g. studied together, co-existance, homology, etc.)

- The purpose of the present study was to examine whether cAMP dependent protein kinase  **PKA**  was implicated in the process of long term potentiation  [ **LTP** ]   **LTP**  in the hippocampal afferent fibre system to the prefrontal cortex in vivo.  Ref: 9786225 Eur J Neurosci, 1998
- Rapid increase in  **PKA**  activity during long term potentiation  [ **LTP** ]  in the hippocampal afferent fibre system to the prefrontal cortex in vivo.  Ref: 9786225 Eur J Neurosci, 1998
- The present experiments examined whether inhibition of protein synthesis,  **PKA** , and MAP kinase activity, treatments that block  **LTP** , also interfere with the consolidation of fear conditioning.  Ref: 10327235 Learn MemLearn Mem, 1998
- We show that there is an NMDA receptor dependent increase in  **PKA**  activity in the prefrontal cortex, only at five minutes after  **LTP**  induction.  Ref: 9786225 Eur J Neurosci, 1998
- In agreement with this hypothesis we found that application of the AC inhibitor SQ 22536 by itself did not affect the early phase of  **LTP** , but did produce a reduction if the cAMP pathway was already attenuated by the  **PKA**  inhibitor H89.  Ref: 10844013 J Neurosci, 2000
- A late phase of  **LTP**  occurring 3 5 hours after stimulation and depending on transcription, protein synthesis and cyclic AMP dependent protein kinase protein kinase A, or  **PKA**  has been described, but it is not known whether transcription of presynaptic and or postsynaptic genes is required to support late phase  **LTP** .  Ref: 9716131 Nature, 1998
- The specific cAMP dependent protein kinase  **PKA**  inhibitor Rp cyclic adenosine monophosphate Rp cAMPS, 25 microM had no effect on the anoxic  **LTP** .  Ref: 9855311 Neuroreport, 1998
- Thus, altering both the total amount of imposed synaptic activity and the temporal spacing between bursts of imposed activity may subtly modulate the  **PKA**  dependence of hippocampal  **LTP**  by engaging distinct isoforms of  **PKA** .  Ref: 11110804 J Neurophysiol, 2000
- Our results show that, like non theta  **LTP** , theta induced  **LTP**  in area CA1 of the mouse hippocampus also involves transcription, translation, and  **PKA**  and suggest that cAMP mediated gene transcription may be a common mechanism responsible for the late phases of  **LTP**  induced by both theta and non theta patterns of stimulation.  Ref: 10456066 Learn MemLearn Mem, 2000
- Consistent with an involvement of cAMP and  **PKA** , activation of adenylyl cyclase induced a synaptic enhancement that was associated with a decrease in PPF and occluded  **LTP** .  Ref: 10516326 J Neurosci, 1999
- Dopamine D 1 D 2 or adenosine A 2A A 1 receptor activation must facilitate  **LTP**  LTDi LTD LTPi due to an augmenting lowering  **PKA**  activity.  Ref: 11084240 BiosystemsBiosystems, 1999
- The present experiments examined whether intra LA administration of inhibitors of protein synthesis or protein kinase A  **PKA**  activity, treatments that block  **LTP**  in hippocampus, interfere with memory consolidation of fear conditioning.  Ref: 10974093 J Neurosci, 2000
- Inhibiting  **PKA**  does not impede tetanus induced  **LTP** , and chelating postsynaptic calcium with BAPTA does not block DEP, indicating that the initial steps of the induction processes are independent.  Ref: 10024349 J Neurosci, 1999
- Using a biochemical approach, we measured  **PKA**  activity at different times after induction of  **LTP** .  Ref: 9786225 Eur J Neurosci, 1998
- Although it is widely agreed that cyclic AMP is necessary for the full expression of long term potentiation  [ **LTP** ]  of synaptic strength, it is unclear whether cyclic AMP or cyclic AMP dependent protein kinase  **PKA**  play roles in the induction of long term depression LTD.  Ref: 10482771 J Neurophysiol, 1999
- We hypothesized that presynaptic  **PKA**  activation is required for  **LTP**  induction but that drugs applied to the granule cell soma cannot diffuse to the terminal within this timescale.  Ref: 10575019 J Neurosci, 1999
- Although bath application of  **PKA**  inhibitor drugs KT5720, Rp 8CPT cAMP S blocked  **LTP**  induction in granule cell Purkinje neuron pairs in culture, selective application to granule cell or Purkinje neuron somata via patch pipettes did not.  Ref: 10575019 J Neurosci, 1999
- Can distinct patterns of synaptic activity induce forms of  **LTP**  that require different isoforms of  **PKA**  ? To address this question, we used transgenic mice that have genetically reduced hippocampal  **PKA**  activity, and a specific pharmacological inhibitor of  **PKA** , Rp cAMPS.  Ref: 11110804 J Neurophysiol, 2000
- We have studied the mechanisms underlying mossy fiber LTD and present evidence that it is triggered, at least in part, by a metabotropic glutamate receptor mediated decrease in adenylyl cyclase activity, which leads to a decrease in the activity of the cAMP dependent protein kinase  **PKA**  and a reversal of the presynaptic processes responsible for mossy fiber  **LTP** .  Ref: 9808469 Neuron, 1998
